# “They say I’m crazy, but I’ve lived through hell.”

**DOI:** 10.1192/j.eurpsy.2024.1089

**Published:** 2024-08-27

**Authors:** N. Molina Pérez, J. Pereira López, M. I. Santana Ortiz, P. Rivero Rodríguez, A. R. N. Del Rosario, M. M. Grimal, V. Acosta Pérez

**Affiliations:** Psiquiatría, Hospital Universitario de Gran Canaria Doctor Negrín, Las Palmas de Gran Canaria, Spain

## Abstract

**Introduction:**

Migration has been present in the evolution of human beings throughout history. Economic inequalities give rise to a permanent flow of people trying to improve their lives. In addition, there are people who are forced to seek asylum or refuge due to wars or political violence. Therefore, the migratory flow, gives rise to a clinical scenario in which, the arrival of immigrant people demands an adaptation of the psychiatric paradigm.

**Objectives:**

The objective of this paper is to review the international scientific literature published on the impact of the migration process on mental health.

**Methods:**

We propose a review of the international scientific literature published in recent years on psychiatry and migration.

We present the case of a 27-year-old male, diagnosed with paranoid schizophrenia, who arrived in the Canary Islands after a 2-year migration process from his country of origin (Senegal).

**Results:**

The limits between normality and pathology of certain types of behavior vary from one culture to another.

In the case of a patient with a mental disorder who has undergone a migration process, an approach based on the cultural formulation of the case should be made, taking into account the process of adaptation to the culture of the host country, as well as the impact of the culture of origin on the patient’s interpretation of his or her psychopathology.

**Conclusions:**

Culture can influence the acceptance or rejection of a diagnosis and treatment, affecting the course of the disease and recovery.

Therefore, understanding the cultural context in which the disease is experienced is essential for a good diagnostic evaluation and effective clinical management.

**Disclosure of Interest:**

N. Molina Pérez: None Declared, J. Pereira López: None Declared, M. I. Santana Ortiz: None Declared, P. Rivero Rodríguez: None Declared, A. R. Del Rosario Grant / Research support from: Jansen Pharmaceuticals, Inc., Consultant of: Jansen Pharmaceuticals, Inc.; Lundbeck, Inc., Employee of: Universidad de Las Palmas de Gran Canaria, Speakers bureau of: Jansen Pharmaceuticals, Inc.; Lundbeck, Inc.; Otsuka Pharmaceutical Co.; Pfizer Inc.; Esteve Pharmaceuticals, S.A.; AstraZeneca Pharmaceuticals LP.; Angelini Pharma S.L.U.; Laboratorios Farmacéuticos ROVI SA., M. Grimal: None Declared, V. Acosta Pérez: None Declared

